# Supplementary figures and images for: Identification and validation of poly-metabolite scores for diets high in ultra-processed food: An observational study and post-hoc randomized controlled crossover-feeding trial
Source: PLoS Med. 2025 May 20;22(5):e1004560. doi: 10.1371/journal.pmed.1004560 (PMC12091781; doi:10.1371/journal.pmed.1004560)

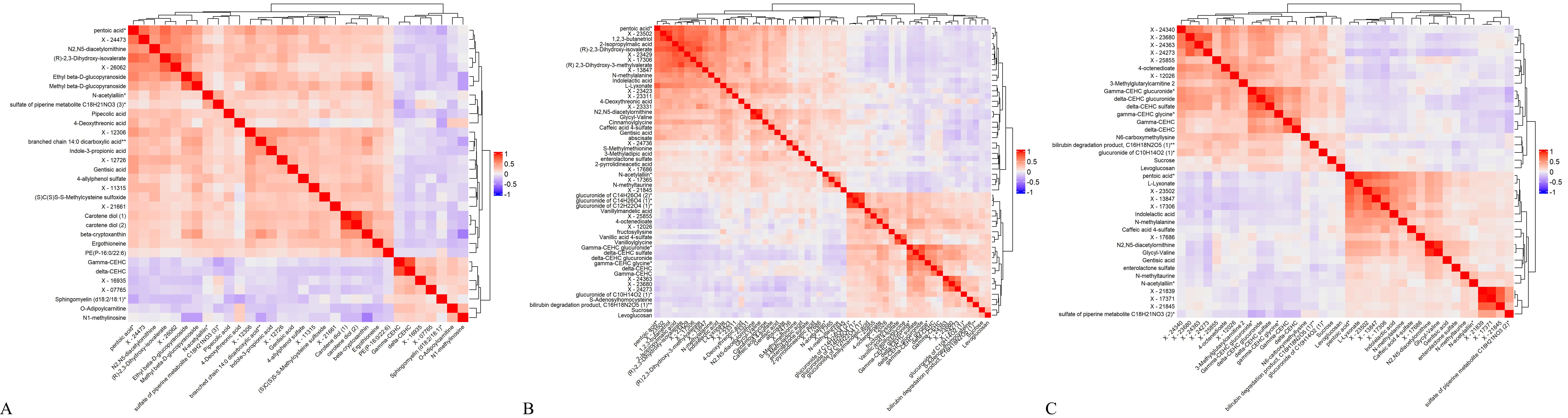

Supplement: S1 Fig — Metabolite-UPF intake (% energy) correlations were estimated using Spearman partial correlation, adjusted for age (continuous), sex (men, women), smoking (cotinine detected: yes, no), race (White, non-Hispanic, African American, Asian, Hispanic) and BMI (18.5 to <25, 25 to <30, 30 to <40, ≥40 kg/m2) in the IDATA Study (N = 718). Multiple testing was corrected for using the Benjamini–Hochberg method; statistical significance was defined as a corrected p-value of <0.01. Correlations between metabolites with |r| ≥ 0.20 and FDR-corrected p-value <0.01 are visualized in the heatmap. The dark purple color shows strong negative metabolite-metabolite correlations and the dark red color shows strong positive metabolite-metabolite correlations. The dendrogram uses hierarchical clustering and shows that metabolites generally cluster according to the direction of their correlation with UPF intake. Abbreviations: UPF = ultra-processed food. (TIFF) [file pmed.1004560.s001.tiff]

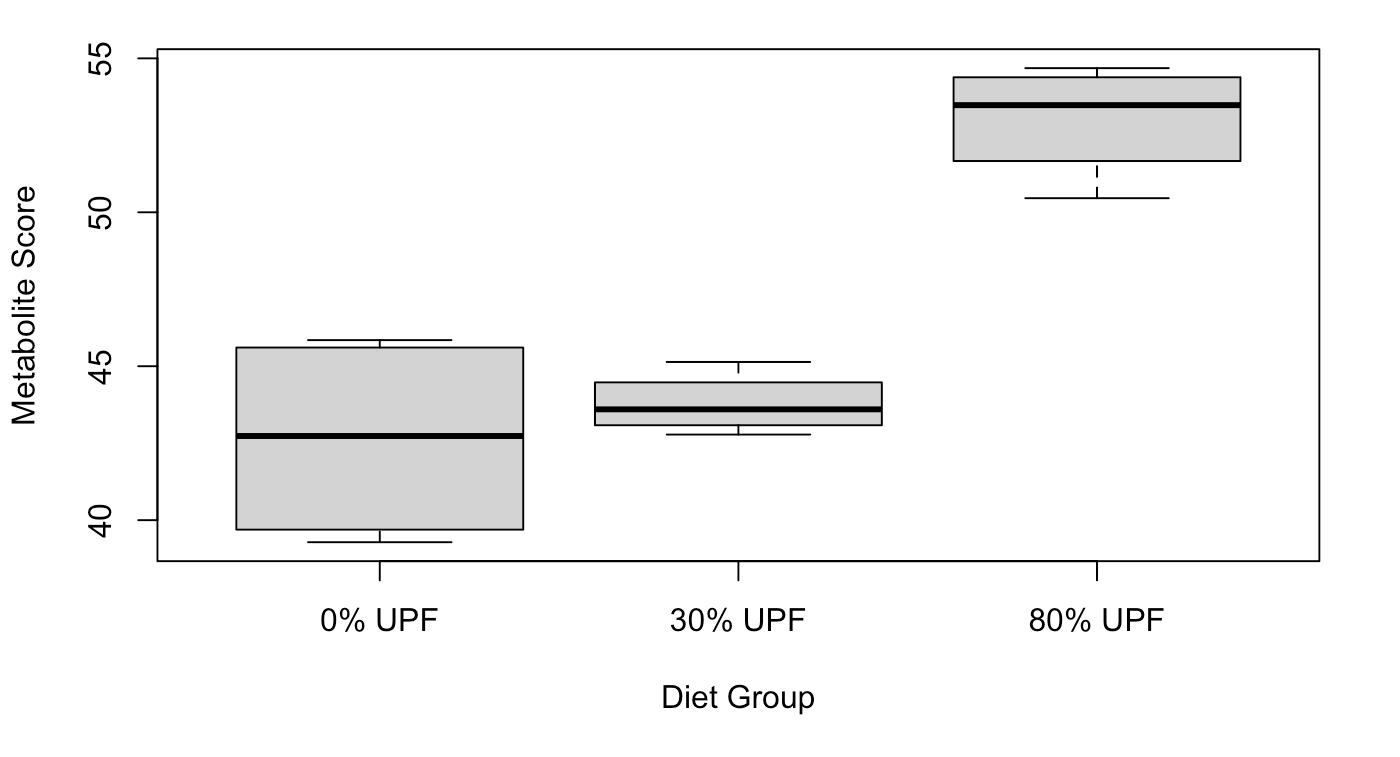

Supplement: S3 Fig — Box plots show the distribution of poly-metabolite scores, developed in the IDATA study using serum, and generated using EDTA plasma from domiciled healthy participants (n = 4) who completed two independent, randomized, controlled, crossover-feeding trials. In the first trial, participants consumed ad libitum, for 2 weeks each, an UPF-DP (i.e., with 80% energy from UPF) and an UN-DP pattern (i.e., 0% energy from UPF). In the second trial, participants, consumed ad libitum a plant-based low-fat and an animal-based ketogenic dietary pattern that were low-carbohydrate/low-fat and contained 30% energy from UPF. The box represents the interquartile range, the center line represents the median, the whiskers show the minimum and maximum values within 1.5 times the interquartile range, and data points outside the whiskers show poly-metabolite score outliers. An ANOVA test (P-value < 0.001) was conducted to compare the mean poly-metabolite scores across the three diet phases with paired samples, and paired t-tests were performed to compare the mean poly-metabolite scores between 0% and 30% UPF (P-value = 0.60) as well as between 30% and 80% UPF (P-value < 0.01). Abbreviations: UN-DP = unprocessed food dietary pattern, UPF-DP = ultra-processed food dietary pattern. (TIFF) [file pmed.1004560.s003.tiff]

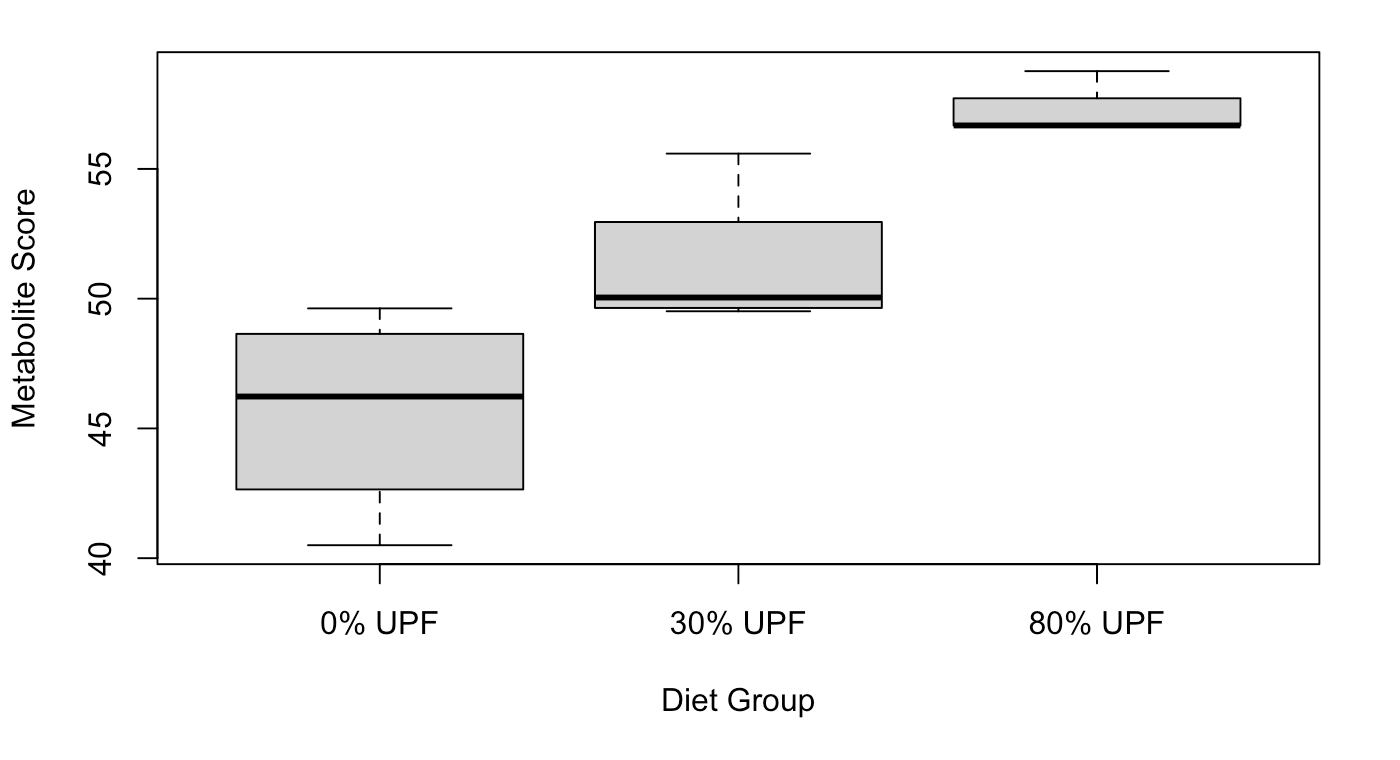

Supplement: S4 Fig — Box plots show the distribution of poly-metabolite scores, developed in the IDATA study using 24-h urine, and generated using 24-h urine from domiciled healthy participants (n = 4) who completed two independent, randomized, controlled, crossover-feeding trials. In the first trial, participants consumed ad libitum, for 2 weeks each, an UPF-DP (i.e., with 80% energy from UPF) and an UN-DP (i.e., 0% energy from UPF). In the second trial, participants, consumed ad libitum a plant-based low-fat and an animal-based ketogenic dietary pattern that were low-carbohydrate/low-fat and contained 30% energy from UPF. The box represents the interquartile range, the center line represents the median, the whiskers show the minimum and maximum values within 1.5 times the interquartile range, and data points outside the whiskers show poly-metabolite score outliers. An ANOVA test (P-value = 0.001) was conducted to compare the mean poly-metabolite scores across the three DPs and paired t-tests were performed to compare the mean poly-metabolite scores between 0% and 30% UPF (P-value = 0.06) as well as between 30% and 80% UPF (P-value = 0.04). Abbreviations: UN-DP = unprocessed food dietary pattern, UPF-DP = ultra-processed food dietary pattern. (TIFF) [file pmed.1004560.s004.tiff]

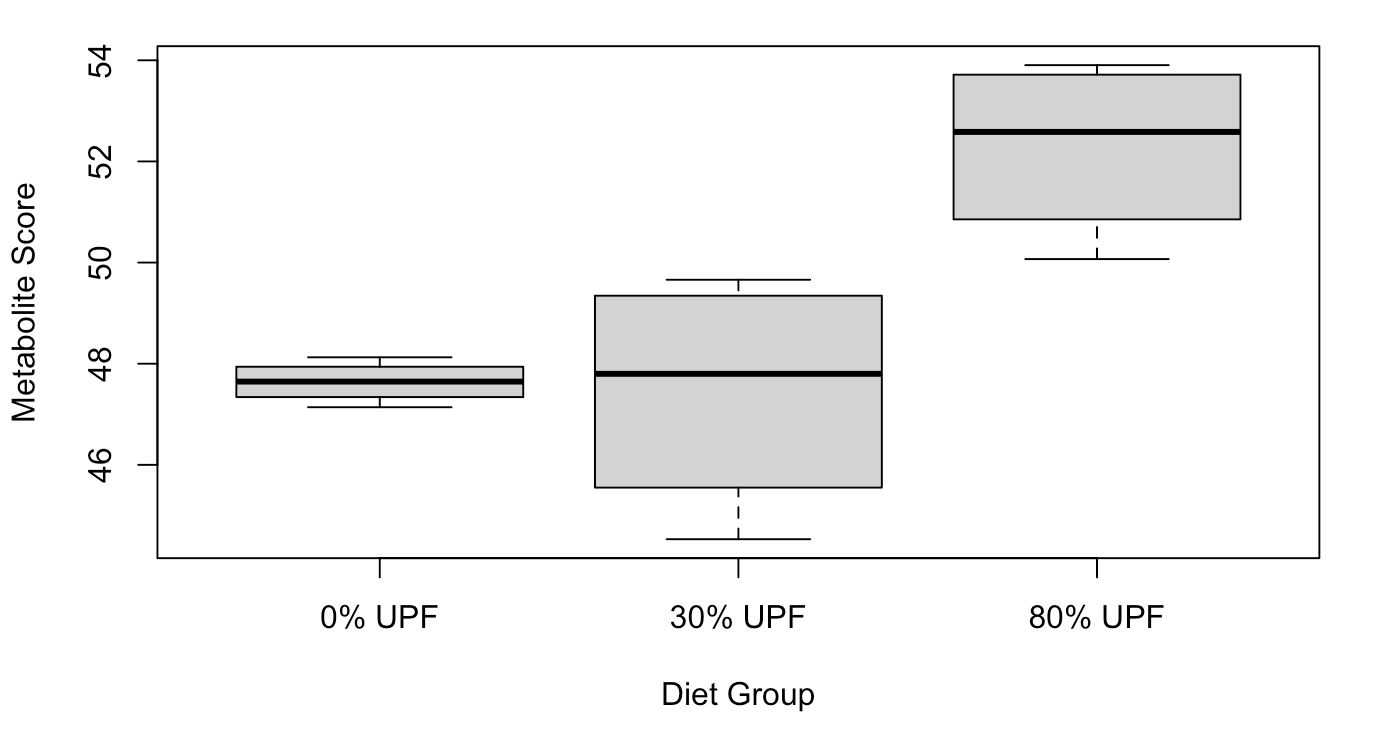

Supplement: S5 Fig — Box plots show the distribution of poly-metabolite scores, developed in the IDATA Study using FMV urine, and generated using spot urine from domiciled healthy participants (n = 4) who completed two independent, randomized, controlled, crossover-feeding trials of. In the first trial, participants consumed ad libitum, for 2 weeks each, an UPF-DP (i.e., with 80% energy from UPF) and an UN-DP (i.e., 0% energy from UPF). In the second trial, participants, consumed ad libitum a plant-based low-fat and an animal-based ketogenic dietary pattern that were low-carbohydrate/low-fat and contained 30% energy from UPF. The box represents the interquartile range, the center line represents the median, the whiskers show the minimum and maximum values within 1.5 times the interquartile range, and data points outside the whiskers show poly-metabolite score outliers. An ANOVA test (P-value < 0.01) was conducted to compare the mean poly-metabolite scores across the three DPs and paired t-tests were performed to compare the mean poly-metabolite scores between 0% and 30% UPF (P-value = 0.90) as well as between 30% and 80% UPF (P-value = 0.02). Abbreviations: FMV urine = first morning void urine, UN-DP = unprocessed food dietary pattern, UPF-DP = ultra-processed food dietary pattern. (TIFF) [file pmed.1004560.s005.tiff]
