# Supplementary material for: Identification and validation of poly-metabolite scores for diets high in ultra-processed food: An observational study and post-hoc randomized controlled crossover-feeding trial
Source: PLoS Med. 2025 May 20;22(5):e1004560. doi: 10.1371/journal.pmed.1004560 (PMC12091781; doi:10.1371/journal.pmed.1004560)
Supplement: Attachment — Submitted filename: PMEDICINE-D-24-02724R1_review.docx [file pmed.1004560.s018.docx]

PMEDICINE-D-24-02724R1

Serum and urine metabolomic profiles of long-term ultra-processed food intake in the

longitudinal Interactive Diet and Activity Tracking in AARP (IDATA) Study

In this study, Abar and colleagues tried to discover intake biomarkers of ultra-processed food in the participants’ serum and urine samples and replicated the the intake prediction based on such panel of discovered biomarkers in an independent set of samples from 20 participants of an intervention study. While finding a set of biomarkers for ultra-processed foods is important, there is something the authors need to clarify. Previous studies usually used randomized, controlled trial (RCT). as the amount of intake is fully controlled and free from any reporting or recall bias commonly occurred in a cohort study (for example, Willis et al 2020 Front Nutr doi 10.3389/fnut.2020.561010). The replication could then be done in a free-living cohort study with habitual diets (as suggested by Maruvada et al 2020 Adv Nutr doi 10.1093/advances/nmz075). Another option is to split the original cohort to a training and a test set, where the training set was used to discover the set of biomarkers, and then the predicted intake was tested against an independent subset of the same cohort (test set, for example, Li et al, 2022 AJCN doi 10.1093/ajcn/nqac094). The authors, however, used the large cohort as a discovery study with > 700 participants and test the derived biomarkers in a much smaller RCT with 20 participants. While there is no strict protocol on how to derive a set of intake biomarkers, did the authors have any special reason to use such a not-so-common “reversed” approach? And could this be also the reason for the observed variability (page 15, Supplementary Table 7)? This selection of methods needs to be justified either in the methods or in the discussion section.
