# Supplementary material for: Identification and validation of poly-metabolite scores for diets high in ultra-processed food: An observational study and post-hoc randomized controlled crossover-feeding trial
Source: PLoS Med. 2025 May 20;22(5):e1004560. doi: 10.1371/journal.pmed.1004560 (PMC12091781; doi:10.1371/journal.pmed.1004560)
Supplement: Attachment — Submitted filename: Response_To_ReviewersComments.docx [file pmed.1004560.s020.docx]

**Author reply:**

Thank you for reviewing our manuscript and providing valuable feedback. We have carefully considered your comments and modified the manuscript accordingly. Your input has greatly improved our study. Please see below the detailed responses and corresponding modifications in the manuscript.

---------------------------------------------------------------------------------------------------------------------
**Comments from the academic editor:**

The most important strength of this study is that the authors identified metabolite predictors of UPF and tested its utility in the cross-over RCT, combining their observational work with the independent RCT data. However, the RCT requires clarification and improved statistical reporting.

**Response:**

Thank you for acknowledging the strength of our multistage design. In response to the reviewers' requests for additional details on the statistical reporting for the randomized, controlled, cross-over feeding trial, we have expanded the Methods section (see pages 12-13, lines 266-286in the clean version) and clarified points of confusion in the results.

UPFs are known to be misclassified. Healthy items, such as mass-produced wholegrain snacks, can be UPF. The authors should analyze and document what food items were predominant in their analyses.

**Response:**

UPFs include a wide range of items including foods generally regarded as “healthy” like wholegrain crackers, wholegrain breakfast cereals, and wholegrain bread as well as foods like sugary breakfast cereals and white bread. Categorizing foods as Nova category 4, or UPF, based on nutrient profiles or health claims would indeed result in substantial misclassification. A major strength of the current study is that we linked 24-hour dietary recall data to USDA FNDDS food codes, which were then disaggregated so that Nova classification was accomplished using food and standard reference, or SR, codes through linkage to the Nova database that was developed by Martinez-Steele et al using National Health and Nutrition Examination Survey (NHANES) 24-hour dietary recall data. In total, 3044 foods were reported by the 718 IDATA participants for 4050 days of intake (mean 5.6 days of intake per person). Of the 3044 corresponding food codes, 2320 contributed, at least in part, to energy intake from UPF. Thus, IDATA covers a wide range of diets and each FNDDS food code contributes a relatively small percentage to percentage energy from UPF at the population level. The top 10 contributors accounted for about 12% of energy intake from UPF (see table below) and include ice cream, white rolls, potato chips, and cola-type soft drinks. We have added the full list of 2320 food codes and how much each contributed to energy intake from UPF in the overall study in **Supplementary Table 1**.

|  | **Food code** | **Food description** | **Nova4 kcal** | **% energy from Nova4** |
| --- | --- | --- | --- | --- |
| 1 | 13110100 | Ice cream, regular, flavors other than chocolate | 85008.43 | 1.91962 |
| 2 | 51150000 | Roll, white, soft | 63145.37 | 1.42592 |
| 3 | 71201010 | White potato, chips | 53484.22 | 1.20775 |
| 4 | 51180010 | Bagel | 52071.41 | 1.17585 |
| 5 | 83107000 | Mayonnaise, regular | 51140.27 | 1.15482 |
| 6 | 51601020 | Bread, multigrain | 47657.6 | 1.07618 |
| 7 | 54408010 | Pretzels, hard | 45998.43 | 1.03871 |
| 8 | 14410200 | Cheese, processed, American or Cheddar type | 44077.98 | 0.99535 |
| 9 | 71401030 | White potato, french fries, from frozen, deep fried | 43412.71 | 0.98032 |
| 10 | 92410310 | Soft drink, cola-type | 42893.86 | 0.96861 |

---------------------------------------------------------------------------------------------------------------------**Reviewer #1:**

This research identified serum and urine biomarkers that are associated with consumption of ultra processed foods (UPF), as determined through correlation-based analyses of self-reported dietary data. Identified biomarkers were used to generate a score, that was validated against two small crossover trials with known levels of UPF consumption.
Overall, this manuscript is clear, cogent, and well presented. The authors have argued the importance of the research convincingly and communicated complex ideas around biomarkers and metabolomics well. There are some opportunities to more clearly define and justify their decisions pertaining to statistical analyses and in some cases important modelling details are missing. They may also consider employing more sophisticated or contemporary statistical approaches. In particular, their decision to analyse longitudinal data by averaging first then simply analysing the averages has prompted a number of questions and may be worth revisiting.
Detailed feedback on the manuscript is provided below. My primary focus has been methodological, but I have made a few broader points as well.  All items are major unless indicated otherwise.

**1.General**
1.1 Consider making clear in both the title and abstract that there are tight constraints on the population of participants being researched, notably age. Not referring to this potentially misleads the reader into understanding this to be about, say, all adults.

**Response:**

The abstract has been revised as follows: “**Methods and findings:** IDATA participants (n=718), aged 50-74 years, with serially collected blood and urine and up to six 24-hour dietary recalls (ASA-24s), collected over 12-months, were included in the metabolomics study, which used ultra-high performance liquid chromatography with tandem mass spectrometry to measure >1000 serum and urine metabolites”. See page 2, lines 24-27.

1.2 [MINOR] Given its salience to the paper, consider tweaking the title to incorporate the secondary as well as the primary aim.

**Response:**

Thank you for the suggestion. We have changed the title to: *Identification of Poly-Metabolite Scores for Diets High in Ultra-Processed Food in an Observational Study with Validation in a Randomized Controlled Crossover-Feeding Trial*.

**2.Abstract**
2.1 Please revise based on the provided feedback.

**Response:**

We have added the age range of IDATA participants to the methods section of the abstract, page 2, line 24.

**3.Introduction**
3.1 [MINOR] "The integration of dietary data, measured via self-report using validated assessment tools, with biomarker measures can control confounding, minimize measurement errors, and enhance statistical power in analyses of diet and disease" I understand this is just background, but is it correct? The methods described in the cited papers appear to adjust for measurement error (not "minimise" it) and I'm struggling with the claim that integration of self-report dietary with biomarker data can "control confounding".

**Response:**

Thank you for providing the opportunity for us to clarify. The reviewer is correct that the cited studies demonstrate how biomarker data can be used to “adjust for” measurement error; we have revised the text (page 5, line 90-92) as follows: “*Previous studies have shown that integrating biomarker data with dietary data, measured via self-report using validated assessment tools, can help to adjust for measurement error and improve the precision of diet-disease association estimates, thereby enhancing statistical power*.” We agree that this approach does not “control confounding” as previously stated and have deleted this text.

3.2 "Currently, nutritional metabolomics research in population-based studies with longitudinal assessment of dietary intake, using validated assessment tools, and serial collection of blood and urine, for generating metabolomics data, is lacking." This suggests a longitudinal analysis will be completed as part of this research, but don't think this is what the authors have done. I would argue averaging two or more time points into one then analyzing is not a longitudinal analysis. Further methodological detail provided below.

**Response:**
We appreciate the reviewer’s comments and acknowledge that averaging repeated exposure measures does not fully capture the day-to-day variability in dietary intake. However, our primary objective was variable selection using LASSO, rather than conducting a full longitudinal analysis. Although more sophisticated measurement error models exist to address daily variability in 24-hour dietary recalls, variable selection methods that fully integrate these corrections are not readily available. Given this limitation, we adopted a simple and consistent analytic approach: we calculated the percentage of energy from UPF for each 24-hour recall and averaged these values for each participant, recognizing that this approach may sacrifice some information on short-term variation.

Similarly, we averaged repeated metabolite measures to account for potential biological variability, an approach that has been shown to improve statistical power in previous metabolomics studies *(Sampson, J. N., et al. (2013). "Metabolomics in epidemiology: sources of variability in metabolite measurements and implications."* Cancer Epidemiology, Biomarkers & Prevention, *22(4), 631–640).* As a result, our analysis is based on these averaged measures, and our poly-metabolite score is derived using LASSO variable selection, with the averaged metabolite levels as predictors and the average percentage energy from UPF as the outcome.

In this revision, we have carefully removed any wording that might imply a longitudinal analysis. Additionally, we have clarified our rationale for using averaged values, acknowledging that this choice prioritizes stable long-term dietary intake estimates over capturing short-term fluctuations (see page 11, lines 233-236).

**4.Methods**
4.1 The IDATA study population and design, Dietary data collection, Nova classification for ultra-processed foods (UPF)
4.1.1 These sections are clear and well-articulated.
4.1.2 I note that while participants may have provided up to six ASA-24s, the study entry criteria required only one. The authors have addressed this by averaging ASA-24-derived UPF percentages. This assumes that a patient with one ASA-24 (at a single point in time) is comparable to a patient with many more (over a longer period). If this is correct, then further justification of this approach is required. Also, averaging results from multiple timepoints removes much of the value of having longitudinal data.

**Response:**

As noted on the first line of Page 14, 97% of participants included in our analysis completed four or more ASA-24s, and we have added that only 3 of the 718 participants completed a single ASA-24. To account for random day-to-day variability and improve the stability of our exposure estimates, we averaged repeated measures for both UPF intake and metabolite levels. This approach provides a more reliable estimate of usual dietary intake, which aligns with our goal of identifying potential biomarkers of long-term dietary patterns rather than acute intake.

We recognize that averaging data across multiple time points assumes comparability between participants with fewer versus more ASA-24s records. However, given the high completion rate of multiple dietary recalls in our study, we believe this concern is mitigated. Additionally, since ASA-24s administrations were unannounced and not timed with biospecimen collections, our approach avoids biases that might arise from aligning single dietary recalls with specific metabolite measures.

While alternative statistical methods could account for within-subject variability differently, our primary goal was biomarker selection rather than modeling longitudinal dietary intake trends. To our knowledge, there are no readily available statistical tools that integrate variable selection with repeatedly measured exposures and outcomes in a high-dimensional metabolomics setting. Given this limitation, we adopted an averaging approach, which provides a practical and interpretable solution for identifying robust biomarker signatures associated with long-term dietary intake (see 24, lines 511-517).

4.2 Biological sample collection, Metabolomics analysis, Quality Control (QC)
4.2.1 I am not a lab scientist so I cannot talk to the veracity of the science in these sections, but I did find them well-written and relatively understandable.
4.2.2 [MINOR] Consider moving the QC results to the Results section.

**Response:**

We have modified the text, accordingly. Please see page 14, lines 310-316.

4.2.3 With respect to the QC results, six-month temporal ICCs appear low suggesting differences in metabolite profiles over time. This finding suggests simply averaging metabolite profiles over time is potentially misleading e.g., high at one time point and low at a subsequent time point is assumed equivalent to medium at both times.

**Response:**

Thank you for raising this important point. Six-month temporal ICCs vary widely across metabolites, and prior methods work has demonstrated that averaging repeated metabolite measures improves statistical power and reduces sample size requirements *(e.g., Sampson, J. N., et al. (2013). "Metabolomics in epidemiology: sources of variability in metabolite measurements and implications."* Cancer Epidemiology, Biomarkers & Prevention, *22(4), 631–640)*. This motivated us to measure metabolite levels at two time points rather than one as is typical of most observational studies. Given our focus on long-term average UPF-metabolites associations, this approach allowed us to reduce the influence of random short-term fluctuations while maintaining consistent intake and metabolite estimates for correlation and LASSO regression analyses.

4.2.4 Following on from this, was the possibility of using multiple pairings of biospecimens with ASA-24-derived UPF proportions instead of just averaging across the total follow-up time then analysing averages?

**Response:**

Blood and urine samples were collected at two fixed time points, six months apart, for each participant. Although these collections sometimes occurred in the same month as an ASA-24s administration, they were not designed as paired assessments. Biospecimen collections were scheduled, whereas dietary recalls (ASA-24s) were unannounced and captured intake over the previous 24 hours. As a result, individual dietary recalls could not be reliably matched with specific biospecimen measurements.

Furthermore, metabolites exhibit varying half-lives, and detailed pharmacokinetic information for many is lacking. This variability would complicate any attempt to pair specific biospecimen measurements with corresponding ASA-24-derived UPF proportions. Given these constraints, our primary objective was to identify a stable metabolite signature associated with long-term high UPF intake, rather than to capture day-to-day variability. Averaging repeated measures provided a more robust estimate of usual intake and metabolite levels under the current study design.

We have added the following text to the discussion: “*Future studies, including feeding trials, with precise pairing of dietary intake and biospecimen collection are needed to better capture temporal dynamics*.” (see page 23, lines 509-510).

**4.3 Statistical analysis**
4.3.1 I am unclear why a Kolmogorov-Smirnov test is required here. Please justify and ensure results are presented and discussed (or remove).

**Response:**

The Kolmogorov-Smirnov test has been removed. We have updated the manuscript accordingly.

4.3.2 Why wasn't alpha controlled for the multiple Kruskal-Wallis rank sum tests, noting 21 separate p-values presented in Table 2?

**Response:**

We have now applied the Benjamini-Hochberg (BH) method to control the False Discovery Rate (FDR) for the 21 p-values in Table 2, to correct for multiple Kruskal-Wallis tests in table 2 and have updated the footnote. Please see Table 2.

4.3.3 Why was a correlation-methods based approach used generally and why was partial Spearman rank correlation used specifically? I am not saying these are incorrect, rather these are not the only choices for these types of analyses (c.f., DOIs 10.1007/s11306-015-0823-6 and 10.1186/s12859-023-05383-0) and some justification should be provided.

**Response:**

Thank you for the opportunity to clarify our methods. In our study, thousands of metabolites were measured in both serum and urine, and we used a correlation-based approach as an initial exploratory analysis to facilitate comparisons with previous observational studies (e.g., *Su D, (2023). “Metabolomic Markers of Ultra-Processed Food and Incident CKD. Clin J Am Soc Nephrol”. 18(3):327-336.* We specifically selected partial Spearman rank correlation because it is robust to non-normal distributions and outliers, which are common in metabolomics data. For this reason, Spearman rank correlations are commonly estimated in epidemiologic studies to estimate metabolite-phenotype associations (*Temprosa M,et al. (2022). Am J Epidemiol. 191(1):147-158).* This method also allows for adjustment of potential confounders such as age and sex, which may influence both dietary intake and metabolite levels. Although alternative approaches exist, we chose partial Spearman correlation due to its suitability for our data characteristics and the exploratory nature of our analysis.

4.3.4 [MINOR] Suggest providing appropriate methodological citations for statistical methods including Benjamini-Hochberg method and LASSO regression.

**Response:**

We have added citations for both methods. Please refer to references 37 and 39 cited on page 11 and 12, lines 246 and 256 in the “Statistical analysis” section.

4.3.5 The methodological detail for LASSO regression is good but some important things are not yet sufficiently clear. What precisely is the outcome being analysed - ordered quintiles like above or something else like actual proportion of energy derived from UPFs? Based on the outcome chosen, what statistical model is used to relate metabolites to UPFs? How are individual metabolites being operationalised - binary presence or absence based on a predetermined level? Actual metabolite levels? Put simply, what is the structure of the regression model that is used with LASSO to select candidate metabolites? Note this detail could be provided in the supplementary materials if word count is an issue.

**Response:**

Thank you again for allowing us the opportunity to clarify our approach. In our analysis, the outcome variable is the percentage of energy derived from UPF, treated as a continuous measure. All metabolite values are also treated as continuous variables and are natural log-transformed to reduce skewness. This operationalization is consistent with our correlation analyses.

Specifically, we applied LASSO variable selection to identify candidate metabolites that predict UPF intake. We used LASSO regression with 10-fold cross-validation to select the optimal penalty parameter (λ). We divided our dataset into training (80%) and testing (20%) subsets, fitting the LASSO model on the training data and evaluating its predictive performance on the testing set using metrics such as RMSE and R-squared. Additionally, we assessed the stability of metabolite selection by repeatedly applying LASSO on randomly selected 80% subsets (100 repetitions) and recording the frequency of selection for each metabolite. See pages 12, lines 255-265.

4.3.6 Relatedly, what exactly is the "poly-metabolite score"? I think it may be the predicted proportion of energy derived from UPFs? If this is correct, I would drop the use of "score" completely and just say "predicted proportion of UPF energy" or similar. If the score is something else, please provide detail on how it is calculated and justification regarding why it was chosen. Either way, ensure it is referred to logically in the rest of the manuscript.

**Response:**

We have added details on how the poly-metabolite score was calculated: “*We calculated UPF poly-metabolite scores, for EDTA plasma, 24-hour urine, and spot urine metabolomics data that was previously generated in a randomized, controlled, crossover-feeding trial [5], as a linear combination of LASSO selected metabolite measures, weighted by corresponding regression coefficients from the LASSO regression models for serum, 24-hour urine, and FMV urine in IDATA, respectively.*” See pages 12-13, lines 267-271.

4.4 UPF poly-metabolite score applied to randomized, controlled, crossover-feeding trial
4.4.1 The use of paired sample t-test in this way is unfamiliar to me. Please provide justification for this choice with appropriate methodological citation(s). One alternative approach is to take the outcome to be predicted as binary (high vs. low UPF) and use an appropriate statistical model with the "UPF poly-metabolite score" as a fixed effect and accounting for paired samples. Also consider a more structured, formal approach to validation e.g., DOI 10.1093/eurheartj/ehu207
4.4.2 Either way, please articulate more clearly how you combined the analyses of the 20- and 4- patient groups.

**Response:**

In our study, we analyzed data from 20 patients using a paired t-test to compare the poly‐metabolite score between the two diet phases. The paired t-test evaluates if the mean difference between paired measurements is zero. It is particularly well‐suited for our cross‐over design because each subject serves as his or her own control, allowing us to directly compare the continuous outcome between paired measurements. Accordingly, “paired, two-sided t-tests were used to compare the diet groups” to test the primary hypotheses in the original trial publication by Hall et al. (*Hall KD, et al. (2019). “Ultra-Processed Diets Cause Excess Calorie Intake and Weight Gain: An Inpatient Randomized Controlled Trial of Ad Libitum Food Intake”. Cell Metab. 30(1):67-77.*). Please refer to page 13.

Regarding the two patient groups, there were 4 subjects who participated in both the ultra-processed diet feeding trial as well as a second feeding trial in which the 30% of energy derived from UPF. First, we calculated the poly‐metabolite score difference for each of the 20 subjects across the two diet phases (0% and 80% energy from UPF). Second, we limited the dataset to the 4 subjects who participated in both feeding trials, and we calculated the poly‐metabolite score difference for each of the 4 subjects across the three diet phases (0%, 30%, and 80% energy from UPF). Additional details have been added for clarity on page 13, lines 272-286.

**5.Results**
5.1Participant characteristics
5.1.1 [MINOR] "Average daily fiber density was also lowest (7.9 g/1,000 kcal) among those in the highest versus the lowest (10.7 g/1,000 kcal) quintile of average UPF intake (p-value<0.001)." Take care with wording here - this could be taken to be a p-value for the contrast comparing the lowest and highest quintiles, whereas I believe the p-value presented is for difference collectively across all quintiles. Consider this for all results when discussing constituent parts tested by an "overall" statistical test.

**Response:**

The reviewer is correct that these p-values are for overall, and not contrast, tests. We have revised the description to avoid confusion: “*Average daily fiber density varied across quintiles of UPF intake (p-value < 0.001), with those in the lowest quintile consuming an average of 7.9 g/1,000 kcal and those in the highest consuming an average of 10.7 g/1,000 kcal. Similarly, the average dietary intake of several micronutrients, including vitamins A, C, D, and E, as well as iron, zinc, potassium, magnesium, phosphorus, and calcium, varied significantly across quintiles of UPF intake (all p-values < 0.05). In contrast, individuals in the highest quintile of UPF intake tended to consume a greater percentage of energy from carbohydrates, added sugars, and saturated fat (all p-values < 0.05;* ***Table 2****)”.* See page 14, lines 298-309.

5.2Metabolite correlations with UPF intake
5.2.1 "(r ≥0.20 & r ≤-0.20, p.FDR ≤ 0.01)" is taken to be "strongly correlated" in several places. This seems to conflate two different things: strength of correlation (r) and strength of the evidence (p). Generally speaking, r = +/- .2 is not "strongly correlated" even with a small p-value. Consider aligning your wording regarding strength of correlation with generally accepted boundaries or justifying your thresholds. Suggest reflecting these definitions in the Methods.

**Response:**

Thank you for raising this important point. We agree that separating the strength of the correlation from statistical significance is warranted, and we have clarified this in the manuscript. The metabolites included in the figures are those that exceeded our threshold of r ≥ |0.20| and an FDR-corrected p-value ≤ 0.01. The text on page 15, lines 335, has been revised to “*Correlation matrices include metabolites with r ≥ |0.20| and an FDR-corrected p-value ≤0.01 (Supplementary Figure 3)*”.

**5.3 Pathway analysis**
5.3.1 So these seven pathways each had combined FDR-corrected p-values<= 0.01 for all three biospecimen types? Consider clarifying.

**Response:**

The Metabolon-defined “super-pathway”, designates seven chemical classes that are measured in many biospecimen types, including blood and urine samples in the current analysis. Each chemical class (e.g., amino acids, lipids, etc.) had a combined FDR-corrected p-value ≤0.01 in serum, 24-hour urine, and FMV urine. Please refer to page 16, line 362.

5.3.2 Why only consider pathways common across all biospecimen types where all other presented analyses treat biospecimen types separately?

**Response:**

Thank you for the opportunity to clarify our methods. Each of the seven super pathways, or broad chemical classes, was represented in blood and urine, though the individual metabolites may have differed. For example, there were more “xenobiotics” measured in urine than in serum. In contrast, not all Metabolon-defined sub-pathways, which reflect more specific metabolic pathways, were represented in both blood and urine. For example, the sub-pathway “Vitamin A Metabolism”, which included 3 metabolites that were significantly correlated with the UPF, was present in serum but not in urine. We ran analyses for all metabolites and corresponding super- and sub-pathways but owing to the large number of findings in IDATA, we focused the text on those that overlapped between serum and urine. Complete pathway results, using Metabolon-defined pathways, are, however, reported for each biospecimen type in **Supplementary Table 4**. We have included additional details in the revised text. Please refer to pages 16-17.

*“Across the three biospecimens, strong and consistent associations with UPF intake were observed for chemical compounds defined by Metabolon Inc. as xenobiotics derived from “food component/plant” and related to benzoate metabolism as well as for compounds of unknown chemical identity. Additional pathways significantly associated with UPF intake in for serum and urine involved: amino acid metabolism, such as methionine, cysteine, SAM, and taurine metabolism, tyrosine metabolism, urea cycle; arginine and proline metabolism, and leucine, isoleucine, and valine metabolism; lipid metabolism, including fatty acid, dicarboxylate, phospholipid metabolism, and fatty acid metabolism (acyl carnitine), were also significantly associated with UPF intake in both serum and urine samples. Energy metabolism pathways, including the TCA cycle and oxidative phosphorylation, showed moderate but consistent associations across biospecimens, pointing to possible alterations in energy production processes with high UPF intake (Supplementary Table 4).”*

**5.4 Poly-metabolite score generation using IDATA study**
5.4.1 "… correlations between the scores and UPF intake were high (≥0.47) …" Assuming this is p≥0.47, similar to my previous point - I question whether 0.47 is "high" correlation. Suggest justification or rewording.

**Response:**

# In nutritional research a correlation coefficient of 0.40 has been cited as a threshold for a concentration biomarker. A correlation coefficient of 0.47 between self-reported dietary intake and a corresponding biomarker is considered “moderate” while one above 0.5 is considered “strong” (*Landberg et al., (2024). “Dietary biomarkers—an update on their validity and applicability in epidemiological studies”.* Nutrition Reviews*, Volume 82, Issue 9, Pages 1260–1280*). We have revised the text and added a citation for Landberg et al: "*Additionally, correlations between each poly-metabolite score and UPF intake in IDATA training and testing sets (r ≥0.47) were moderate to strong*," as reflected on page 17, line 377-378.

5.5 UPF poly-metabolite score applied to a randomized, controlled, crossover-feeding trial
5.5.1 Aside from the concerns about the approach already raised, please ensure any presented statistics are described in the methods (noting ANOVA p-values presented in the supplementary results).

**Response:**

To clarify our methods, we revised the **Supplementary Figure 4-6** legend as follows: “*An ANOVA test was conducted to compare the mean poly-metabolite scores for 4 subjects across the three diet phases with paired samples, and paired t-tests were performed to compare the mean poly-metabolite scores between 0% and 30% UPF as well as between 30% and 80% UPF”.* Also we revised the text on page 13, lines 283-286 *(****Supplementary Figures 4-6****)”*.

**6.0 Discussion and Conclusion**
6.1 I will not repeat feedback already provided when similar feedback applies to the discussion, but please ensure any changes made in the earlier sections are reflected here too.

**Response:**

The discussion has been revised to reflect changes made to earlier sections of the paper.

6.2 "The positive correlation between N6-carboxymethylysine and UPF intake in our study suggests that a dietary pattern high in UPF may, in part, increase risk of developing type 2 diabetes (46) through this metabolite or related metabolic pathways." Correlation does not imply causation. Please reword.

**Response:**

The text has been revised to: "*The positive correlation between N6-carboxymethyllysine and UPF intake in our study suggests a potential link between a dietary pattern high in UPF and type 2 diabetes risk*." This revision can be found on page 20, lines 437-439.

6.3 "Thus, our novel poly-metabolite scores could be used as an objective and complementary measure of UPF intake in future epidemiological investigations." Two things. Firstly, is this justified or is further validation of the presented "poly-metabolite score" appropriate first? Secondly, much is said in the introduction about combining self-report dietary information and biomarkers to improve accuracy and power (particularly using regression calibration), but this is not revisited in the discussion.

**Response:**

We agree that further validation is necessary to establish the robustness and generalizability of our poly-metabolite score. Controlled feeding trials and observational studies in diverse populations will be essential for refining its predictive value and assessing its broader applicability. Our findings provide an important foundation, demonstrating the potential of multi-metabolite profiles as objective markers of UPF intake, similar to polygenic risk scores, which have been iteratively improved with expanding datasets (see page 23, lines 515-517).

As emphasized in the introduction, integrating self-reported dietary intake with biomarker-based assessments is a valuable approach to improving dietary exposure measurement. Regression calibration and related statistical methods present promising opportunities to reduce measurement error and enhance predictive accuracy. While our primary focus in this study was to develop and evaluate the poly-metabolite score, we now note in the conclusion that “F*uture studies are needed to validate our UPF poly-metabolite scores in more diverse populations with varying diets and to explore whether integrating poly-metabolite scores with self-reported can reduce measurement error and improve risk prediction*.” (see page 24, lines 524-528).

6.4 "Firstly, diet over 12 months was assessed via multiple validated 24-hour dietary recalls, with more than 97% of participants completing four or more. This helped minimize measurement error due to random within-person day-to-day variation in dietary intake, and it also allowed for more accurate and detailed dietary data collection than a food frequency questionnaire." I question the veracity of this strength. As discussed previously, averaging away within patient variability does not necessarily better reflect reality compared with, say, modelling it directly with multiple time points per patient.

**Response:**

As discussed in **Section 4.1.2,** while modeling within-subject variability directly using multiple time points per participant is a valid approach, our primary objective was **biomarker selection rather than characterizing longitudinal dietary intake trends.** To our knowledge, there are no readily available statistical methods that integrate **variable selection with repeated exposure and outcome measurements in a high-dimensional metabolomics setting**. Given this limitation, we adopted an **averaging approach,** which provides a **practical and interpretable strategy** for identifying **robust biomarker signatures associated with long-term dietary intake.** This method helps mitigate the influence of short-term dietary fluctuations while still preserving meaningful associations between biomarkers and habitual dietary patterns. We have noted this in the discussion on pages 23-24 (detailed above).

6.5 The conclusion is succinct, well-justified, and clear. Well done!

**Response**:

Thank you for your supportive feedback.

7.0 **Tables and Figures**
7.1 I will not repeat feedback already provided when similar feedback applies to the tables and figures, but please ensure any changes made in the earlier sections are reflected here too.

**Response**:

We have revised tables and figures accordingly. Specifically, we have updated Tables 1 and 2, moved Figure 1 (showing heatmaps) to the supplementary figures, and replaced it with a new figure illustrating the correlations between UPF intake and metabolites. This new figure presents an analysis of serum, 24-hour urine, and FMV urine metabolites selected by LASSO regression as predictive of UPF, categorized by chemical class and metabolic sub-pathway. Additionally, we have adjusted the resolution and font sizes of all figures to ensure readability and consistency. Finally, we have added **Supplementary Figure 2**, which depicts the design of our IDATA study.

7.2. Some of the figures (main and supplementary) are very detailed and difficult to read. Consider splitting into separate sub-figures and/or ensure vector graphics (e.g., EMF or SVG in Windows) are provided, as they are sharp to arbitrary zoom levels.

**Response:**

We have generated high resolution figures for both the main and supplementary documents.

7.3 Suggest not using exponential notation for p-values

**Response:**

We have made the necessary corrections within the main manuscript text.

7.4 I am not sure what is going on with female vs. male in Figure S2. Please adjust so separation is clear.

**Response:**

We have removed this figure as it is provided little information beyond what could be communicated succinctly in the manuscript.

---------------------------------------------------------------------------------------------------------------------
**Reviewer #2:** The manuscript submitted by Abar and colleagues describes the association of urine and serum metabolomic profiles with ultra-processed food intake in a population of 718 individuals. The manuscript is generally logically and descriptively written and in most places is easy to understand (though with some exceptions). The experimental design is generally appropriate though the population studied was very focused and further studies in different populations will be required to validate in the future. The conclusions derived are appropriate based on the data presented and it was nice to see the conclusions validated in an independent study where data was already available. There are some information and methods not included which should be included and although the strengths of the study were discussed, the weaknesses of the study should also be discussed.

There are a number of aspects which the authors should consider to provide a more descriptive and balanced manuscript.

1. Although supplementary file 1 describes the workflow for choice of participants it is somewhat confusing as to when the questionnaire was completed compared to when the biosamples were collected. This is important because there is an assumption that diet is static across the 12 months and so the biosamples represent the questionnaire-defined diet. A further description to detail this (and possibly a figure) would benefit the reader. Also, discussing this in the limtations would be appropriate.

**Response:**

We have added **Supplementary Figure 2** to illustrate the study timeline, including diet assessments and biospecimen collections. We have also stated on page 6, line 122-124 that “*Our analytic sample included* *the 718 IDATA participants with dietary data, including at least one ASA-24, and available serum, 24-hour urine, and first morning void (FMV) urine, collected at two time points, 6 months apart, for metabolomics analysis (****Supplementary Figure 1 & 2****)*”.

2. Page 7. Blood samples were defined but which type of blood sample (serum or plasma), if plasma what was the anti-coagulant and is serum how was coagulation performed?

**Response:**

For the IDATA study, serum samples were collected and used for metabolomics analysis. For the randomized, controlled, cross-over feeding trial, EDTA plasma was collected and used for metabolomics analysis. Sample type is included in the methods section on page 8, line 166 (IDATA) and page 12, line 267 (trial).

3. The metabolomics methods are poorly described and requiure significant more information.

**Response:**

We have added a detailed description of the metabolomics analysis including citations that further describe the development of the Metabolon Global Discovery Panel. Please refer to pages 8-10. Given the length of the description, we can move this to the supplement at the discretion of the editor.

4. Page 8. The metabolomic methods describe tandem mass spectrometry which suggests that a triple quadrupole mass spectrometer is applied, and that data is reported as concentrations. I believe this is not the case for this service provider and so more details of the instrument model and manufacturer is required. Following on from this, more information on the types of liquid chromatography and methods applied is required.

**Response:**

Metabolon performed the analyses using a Waters ACQUITY UPLC system coupled with a Thermo Scientific Q-Exactive high resolution/accurate mass spectrometer interfaced with a heated electrospray ionization (HESI-II) source and Orbitrap mass analyzer operated at 35,000 mass resolution. Thermo Scientific describes the Q-exactive as a hybrid quadrupole-Orbitrap mass spectrometer.

For UPLC, four methods were used. To accomplish this, sample extract was dried then reconstituted in solvents compatible to each of the four methods. Each reconstitution solvent contained a series of standards at fixed concentrations to ensure injection and chromatographic consistency. One aliquot was analyzed using acidic positive ion conditions, chromatographically optimized for more hydrophilic compounds. In this method, the extract was gradient eluted from a C18 column (Waters UPLC BEH C18-2.1x100 mm, 1.7 µm) using water and methanol, containing 0.05% perfluoropentanoic acid (PFPA) and 0.1% formic acid (FA). Another aliquot was also analyzed using acidic positive ion conditions; however, it was chromatographically optimized for more hydrophobic compounds. In this method, the extract was gradient eluted from the same afore mentioned C18 column using methanol, acetonitrile, water, 0.05% PFPA and 0.01% FA and was operated at an overall higher organic content. Another aliquot was analyzed using basic negative ion optimized conditions using a separate dedicated C18 column. The basic extracts were gradient eluted from the column using methanol and water, however with 6.5mM Ammonium Bicarbonate at pH 8. The fourth aliquot was analyzed via negative ionization following elution from a HILIC column (Waters UPLC BEH Amide 2.1x150 mm, 1.7 µm) using a gradient consisting of water and acetonitrile with 10mM Ammonium Formate, pH 10.8. The MS analysis alternated between MS and data-dependent MS^n^ scans using dynamic exclusion. The scan range varied slighted between methods but covered 70-1000 m/z.

We have added this detail to the methods (pages 8-10) as noted above.

5. Page 8. Osmolarity measurements were performed for urine. Why? No further mention of these data is included in the manuscript. These data would normally be used for pre- or post-analysis normalisation but this is not described.

**Response:**

Osmolarity measurements were performed by Metabolon as detailed on page 9; batch-normalized metabolite data were divided by the osmolarity value. Then each metabolite was re-scaled to have a median of 1, and imputation was performed. We have clarified this in the text on pages 9, lines 182-186 and page 10, lines, 215-217.

6. Page 8 and elsewhere. Proof read the manuscript; for example, micro greek symbol and not u when defining microlitres.

**Response:**

Thank you for catching this typo. All uses of “uL” have been changed to “μL”.

7. Page 8. Fixed concentrations of standards. What are these standards and how are they applied? I assume they are isotopically-labelled internal standards and if so how is the data used to assess quality of the data or report concentrations.

**Response:**

For the Global Discovery Panel, Metabolon uses recovery standards and internal standards for QA/QC. Metabolon reports that a cocktail of QC standards that were carefully chosen not to interfere with the measurement of endogenous compounds were spiked into every analyzed sample, allowed instrument performance monitoring and aided chromatographic alignment. Recovery standards were used to assess variability and verify performance of extraction and instrumentation, and internal standards were used to assess variability and performance of instrument. Instrument variability was determined by calculating the median relative standard deviation (RSD) for the standards that were added to each sample prior to injection into the mass spectrometers. The RSD for QC serum samples was 8%.

Internal standards were not used to estimate concentrations. Rather metabolites peaks were quantified using area-under-the-curve and represent relative levels rather than absolute concentrations. We have checked to ensure that all metabolite measures for IDATA and the feeding trial are referred to as levels and not concentrations.

8. Page 8. How are metabolites identified? MS1, MS/MS, RT, other?

**Response**:

The Metabolon HD4 Panel employs Ultrahigh Performance Liquid Chromatography-Tandem Mass Spectroscopy. Identifications are made by comparing mass-to-charge ratios (m/z), retention index, and mass spectral fragmentation patterns against a reference library of authenticated standards. The retention index (RI) is a key factor, with biochemical identifications based on a narrow RI window corresponding to the proposed identification. Additionally, mass accuracy is ensured through an accurate mass match to the library within a tolerance of ±10 ppm. Confirmation is further supported by comparing MS/MS forward and reverse scores between experimental data and library standards. Thus, metabolite identification is achieved through a combination of MS1 data (accurate mass), MS/MS data (fragmentation pattern matching), and retention index (chromatographic behavior), providing a comprehensive approach to metabolite characterization.

Details on metabolite identification have been added to the methods with relevant citations (see page 10).

9. Page 9. Normalised by run day. What does this mean as it is not something commonly applied in metabolomics. Also how are data normalised and scaled for data analysis?

**Response:**

"Normalized by run day" refers to an adjustment process to reduce variability between analytical runs, ensuring consistency across multi-day analyses. More specifically, each compound was corrected in run-day blocks by registering the medians to equal one (1.00) and normalizing each data point proportionately (termed the “block correction”; see figure below). This has been added to the text, please see page 10.

**Visualization of data normalization steps for a multiday platform run.**

10. Page 11. What definition of normal weight, overweight and obese was used as this can differ between continents?

**Response:**

The text has been revised to “with 27%, 43% and 30% of individuals classified as normal weight (18.5 to <25 kg/m^2^), overweight (25 to <30 kg/m^2^), and obese (> 30 kg/m^2^), respectively”. See page 13, lines 292-293.

11. Page 12 onwards. The authors describe some metabolites as amino acids which in reality are not amino acids and will confuse some readers. The authors should define clearly how these metabolites are classified, i.e. assume based on Metabolon classification system. Some point for 4-deoxythreonic acid which is not a lipid.

**Response:**

We have checked and confirmed the “super” and “sub” pathways assigned by Metabolon in our data file. For the compounds highlighted by the reviewer, we also checked the Human Metabolome Database (HMDB) and found that of the 5 amino acids listed on page 15, 4 were also listed as amino acids on HMDB. The one exception was gentisic acid, which was listed as an organic compound, specifically a hydroxybenzoic acid derivative, on HMDB. We state on page 12 that the super- (i.e., chemical class) and metabolic sub-pathways were assigned by Metabolon. We have maintained these for consistency with other Metabolon studies. However, we also acknowledge that compounds may be involved in a number of metabolic pathways, which would not be reflected by Metabolon’s mutually exclusive classification approach.

12. Page 12. C18H21NO3, all numbers should be subscripted to be correct.

**Response:**

The reviewer is correct. We have corrected this and similar subscript issues throughout the manuscript.

13. Page 14, pathway analysis. The methods applied for pathway analysis should be included in the methods section.

**Response:**

The pathway analysis methods (page 12) and results (page 16-17) have been expanded.

14. The discussion should discuss the origin of the important metabolites. Are these diet-derived, gut microbiome-derived or human responses to dietary components.

**Response:**

While some metabolites have been studied and well characterized in online databases like PubChem and HMDB, as well as in publications, many are not. On pages 20-22, we’ve endeavored to highlight and discuss a number of these metabolites that were correlated with UPF intake and were selected by LASSO regression and included in one or more of the poly-metabolite scores. We’ve cited prior studies linking dietary intake and related exposures to key metabolites. For example, we state that, “*In the serum set, three metabolites in the chemical class, cofactors and vitamins, were negatively correlated with UPF intake; β-cryptoxanthin and carotene diol (1) & (2) are provitamin A carotenoids found in citrus fruits and some vegetables, such as red peppers, that have been identified as candidate biomarkers of fruit and vegetable intake [22, 46]. β-cryptoxanthin has also been associated with adherence to a healthy dietary pattern [22, 46, 52].*” We also discuss how “*Serum levels of N6-carboxymethylysine, an advanced glycation end-product [that was positively correlated with UPF intake], have been associated with diabetes incidence and severity as well as with risk of other cardiometabolic diseases [48-50]*.”

Overall, many of these important metabolites were appear to be derived from diet or reflective of a response to diet. However, some well-know diet-related, microbiome-derived metabolites, such as secondary bile acids and TMAO, were not selected by our models, and some well-known diet-related, microbiome-derived metabolites, like short chain fatty acids, were not measured, owing to their volatile properties. We suspect that some of the unknown metabolites that were selected as important predictors of UPF intake are gut microbiome derived. Identifying these unknown compounds is an important next step in metabolomics research.

15. Figure 1. These heatmaps show correlations between metabolites and other metabolites as well as UPF intake. This makes these figures difficult to view and understand especially with the small size of the text. It would be more appropriate to visualise the corrections between metabolites and UPF food intake only.

**Response:**

The heat map was intended to illustrate how many of the metabolites are correlated with each other and their clustering patterns, but we agree with the reviewer that this is not critical to understanding the main findings. Therefore, we have moved this figure to the supplementary materials and replaced it with a bubble plot, which more clearly and succinctly visualizes key findings including the direction and magnitude of the correlations for each metabolite selected by LASSO regression and included in one of the three poly-metabolite scores. In addition, the bubble plot shows the overlap between the three biospecimen types as well as the Metabolon assigned chemical class and metabolic sub-pathway, providing a more detailed and structured visualization of these associations. Please see **Supplementary Figures 4-6**.

16. Supplementary figure 1 includes errors, some text is cut-off and not visible and u used for micro.

**Response:**

Thank you for making us aware of this error. It has been corrected.

17. The manuscript discussed strengths of the study but not weaknesses. To provide a balanced story both strengths and weaknesses should be discussed.
(i) The population sampled is not diverse in age (all >50 years) and probably educated as access to internet required as well as being predominantly white and non-hispanic.

**Response:**

We have addressed this by stating: "While our IDATA Study population lacked diversity, the participants consumed a range of diets, and diets high in UPF were not the same, suggesting our findings may be generalizable to other populations”. Please see page 23 lines 504-506.

Please also see **Table 1** that shows the percentage energy from UPF, which ranged from 12% to 82% suggesting our findings may be generalizable to more diverse populations.

(ii) The biosamples were collected at different times to the questionnaire data and so there is an assumption that the diet at the time of the questionnaire is the same as when biosamples were collected. As metabolomic profiles are dynamic, if diet changes this will be viewed in the profiles quickly within hours normally.

**Response:**

The reviewer is correct that 24-hour dietary recall data and biospecimen collections were not synchronized (see new **Supplementary Figure 2**). As discussed in responses to the first reviewer, this was by design to reduce the likelihood that participants would alter their dietary intake. To account for day-to-day variability, we averaged daily percentage energy from UPF intake across up to 6 dietary recalls collected over 12 months as well as metabolite measures from samples collected at two time points 6 months a part. Our goal was to characterize to the correlations between average long-term intake and average metabolite levels and develop a poly-metabolite score with predictive utility in observational studies of usual diet and chronic disease risk.

(iii) It is unclear which types of metabolites and lipids were studied. The results predominantly discuss non-lipids and it is unclear whether this is because lipids were not studied or because lipids were studied but where not statistically significant. You would expect lipids to change significantly with UPF.

**Response:**

Thank you for your comment. The metabolomics assay that we used identified 52 and 21 lipid metabolites significantly correlated with UPF in serum and urine, respectively. All measured metabolites, including those classified as lipids were included in correlation (**Supplementary Tables 2-4**) and LASSO analyses ((such as PE (P-16:0/22:6), PE (P-16:0/18:1) & LysoPC(O-18:0)). Owing to a large number of significant correlations, in the main figures and tables, we highlighted a handful of the metabolites with the highest correlations with UPF intake as well as some of those included in poly-metabolite scores. The full results, including details on metabolite chemical class and sub-pathway classification, are included in the **Supplementary Tables 2-4**.

---------------------------------------------------------------------------------------------------------------------Reviewer #3: The manuscript by Abar et al. describes the metabolomic investigation of long-term ultra-processed food intake in the longitudinal cohort called Interactive Diet and Activity Tracking in AARP (IDATA) Study. This large cohort study investigated urine and serum samples of 787 individuals using the Metabolon platform. The authors identified metabolic predictors for the ultra-processed foods (UPFs) using a LASSO regression model, which can be used for determining UPF dietary intake. The study has been performed well and the manuscript is well written. The authors have just missed to discuss their powerful large dataset in more detail, which will improve the impact of their important study covering the medical and nutritional field. In summary, I recommend publication of this study in PLoS Medicine after my remarks have been addressed.

Major remarks:

- The identified metabolites seem to have a high predictive power. To compare the value of their biomarker pattern with other studies, it is important to perform receiver operating characteristics analysis.

**Response:**

Thank you for your suggestion. Instead of using receiver operating characteristic (ROC) analysis, we evaluated the robustness of our biomarker signature by applying it to data from a previously conducted randomized controlled trial (RCT). This approach allowed us to assess its predictive performance in an independent, real-world setting, providing complementary validation.

While ROC analysis is valuable for evaluating classification accuracy, our primary goal was to test the biomarker signature’s ability to predict UPF intake in a controlled population with a well-defined dietary intervention. Given that our outcome is a continuous measure rather than a binary classification, ROC analysis may not fully capture the predictive utility of our biomarkers. In contrast, applying the biomarker signature to an RCT dataset enables us to assess how well it generalizes to external populations. However, we acknowledge that ROC analysis could be useful in future studies, particularly for evaluating biomarker cutoffs or thresholds for UPF intake classification. We appreciate this suggestion and will consider incorporating ROC-based approaches in subsequent work.

- An overview figure to describe the study design of this study and the cohort would be benefit the manuscript.

**Response:**

We have added a figure, **Supplementary Figure 2**, illustrating the design of the study. The caption explains that participants were randomized to one of two timelines to minimize seasonal effects on diet and to facilitate logistics. They completed up to six ASA-24s over 12 months, indicated by blue dots on the timeline, and 718 participants provided biospecimen collections at two time points, 6 months apart, either at month 1 and 6 or month 6 and 12. Because of day-to-day variability in both diet and metabolite measures, we used repeated measures to estimate 12-month average percentage energy from UPF intake using data from up to six ASA-24s as well as 6-month average metabolite levels from two collections taken six months a part.

- The authors describe pathway analysis throughout the manuscript but the discussion on these pathway is very brief. I would like to see a more thorough description of the identified pathways, how they have been linked previously to dietary differences and what can be medically learned from these alterations.

**Response:**

Thank you for your comment. We appreciate the importance of providing a detailed discussion of our pathway findings. However, we have already covered a substantial amount of material in this manuscript, and providing an in-depth explanation of all sub-pathways related to UPF consumption would extend beyond the current scope of this study. Our primary focus was to identify and report significant pathways associated with UPF intake, while offering key insights into their potential biological relevance. We believe that the current level of detail maintains the manuscript's clarity and focus, while leaving room for future studies to explore the specific mechanistic links between these pathways, dietary differences, and medical implications in greater depth.

- This sentence should be moved or summarized in the conclusion section: "Thus, the metabolomic profiles that we identified likely reflect a dietary pattern characterized not only by high UPF consumption but also by low whole food, including fresh fruits and vegetables, consumption."

**Response:**

Thank you. It has been moved to the conclusion section. Please see page 23, line 520-522.

- Figure 1: All text should have the size of the left panel in the first top left heatmap.
- The resolution of Figure 3 must be improved.

**Response:**
Thank you for catching these oversights. We have fixed the size of the text in Figure 1 (now **Supplementary Figure 3-6**), and we have generated a new high-resolution image for all the figures.

Minor remarks:

- "meat eater" is a trivial word and should be replaced by the correct scientific term for this cohort.

**Response:**

It has been revised to “*people who consume meat*”. See page 19, line 421.

- Instersection should be correct to Intersection in the Figure 2 legend.

**Response:**

The text was corrected.

---------------------------------------------------------------------------------------------------------------------Reviewer #4: PMEDICINE-D-24-02724R1
Serum and urine metabolomic profiles of long-term ultra-processed food intake in the
longitudinal Interactive Diet and Activity Tracking in AARP (IDATA) Study

In this study, Abar and colleagues tried to discover intake biomarkers of ultra-processed food in the participants' serum and urine samples and replicated the the intake prediction based on such panel of discovered biomarkers in an independent set of samples from 20 participants of an intervention study.

While finding a set of biomarkers for ultra-processed foods is important, there is something the authors need to clarify. Previous studies usually used randomized, controlled trial (RCT).  as the amount of intake is fully controlled and free from any reporting or recall bias commonly occurred in a cohort study (for example, Willis et al 2020 Front Nutr doi 10.3389/fnut.2020.561010). The replication could then be done in a free-living cohort study with habitual diets (as suggested by Maruvada et al 2020 Adv Nutr doi 10.1093/advances/nmz075). Another option is to split the original cohort to a training and a test set, where the training set was used to discover the set of
biomarkers, and then the predicted intake was tested against an independent subset of the same cohort (test set, for example, Li et al, 2022 AJCN doi 10.1093/ajcn/nqac094). The authors, however, used the large cohort as a discovery study with > 700 participants and test the derived biomarkers in a much smaller RCT with 20 participants. While there is no strict protocol on how to derive a set of intake biomarkers, did the authors have any special reason to use such a not-so-common "reversed" approach? And could this be also the reason for the observed variability (page 15, Supplementary Table 7)? This selection of methods needs to be justified either in the methods or in the discussion section.

**Response:**

We acknowledge that many previous studies have developed dietary biomarkers using randomized controlled trials as the discovery set and then validated them in a free-living cohort. However, our approach was driven by the available data. The large cohort study provided a unique opportunity to identify metabolite signatures associated with UPF intake in a diverse population with repeated dietary assessments. The RCT we used for validation was the only crossover feeding trial we identified that specifically tested UPF intake while also collecting plasma and urine samples. Given that both studies were not originally designed for biomarker development, we made a pragmatic decision to use the cohort for discovery and the RCT for validation.

While this approach is less common, it allowed us to test whether the identified poly-metabolite score could differentiate between controlled high- and low-UPF dietary conditions, minimizing biases inherent in self-reported dietary data. We acknowledge that this study design differs from the conventional method of developing biomarkers in a controlled setting first. However, given the limited availability of UPF-specific feeding trials, this was the most feasible validation approach available to us.

Regarding variability, we agree that the small sample size of the RCT may contribute to the observed variation. To strengthen our findings, we internally validated the poly-metabolite score within the cohort study using LASSO regression with repeated training and testing splits (as described in the Methods section). This approach helped ensure that our biomarker selection was not overly dependent on a single dataset and remained robust across different subsamples. We will clarify these points in the revised manuscript to directly address these concerns (see pages 23 lines 510-518).

Any attachments provided with reviews can be seen via the following link: <https://gcc02.safelinks.protection.outlook.com/?url=https%3A%2F%2Fwww.editorialmanager.com%2Fpmedicine%2Fl.asp%3Fi%3D967715%26l%3DJB1LYYWX&data=05%7C02%7Cleila.abar%40nih.gov%7Cebb2ae5a8c8046e5f64008dd3ac4ac14%7C14b77578977342d58507251ca2dc2b06%7C0%7C0%7C638731338859694814%7CUnknown%7CTWFpbGZsb3d8eyJFbXB0eU1hcGkiOnRydWUsIlYiOiIwLjAuMDAwMCIsIlAiOiJXaW4zMiIsIkFOIjoiTWFpbCIsIldUIjoyfQ%3D%3D%7C0%7C%7C%7C&sdata=fclgdlPSNhwXFNYvQRdXjwAJ7LWa85oJufzwdqsR9cA%3D&reserved=0>
--------------------------------------------------------- ---
General editorial requests:
(Note: not all will apply to your paper, but please check each item carefully)

**Response:**

We have revised the manuscript accordingly and have added the RCT registration number as requested. Please refer to page 6, line 120.

*       We ask every co-author listed on the manuscript to fill in a contributing author statement, making sure to declare all competing interests. If any of the co-authors have not filled in the statement, we will remind them to do so when the paper is revised. If all statements are not completed in a timely fashion this could hold up the re-review process. If new competing interests are declared later in the revision process, this may also hold up the submission. Should there be a problem getting one of your co-authors to fill in a statement we will be in contact. Please do not add or remove authors without first discussing this with the handling editor. You can see our competing interests policy here: <https://gcc02.safelinks.protection.outlook.com/?url=http%3A%2F%2Fjournals.plos.org%2Fplosmedicine%2Fs%2Fcompeting-interests&data=05%7C02%7Cleila.abar%40nih.gov%7Cebb2ae5a8c8046e5f64008dd3ac4ac14%7C14b77578977342d58507251ca2dc2b06%7C0%7C0%7C638731338859707393%7CUnknown%7CTWFpbGZsb3d8eyJFbXB0eU1hcGkiOnRydWUsIlYiOiIwLjAuMDAwMCIsIlAiOiJXaW4zMiIsIkFOIjoiTWFpbCIsIldUIjoyfQ%3D%3D%7C0%7C%7C%7C&sdata=LFmz1ruPwmBTcQ6KVsDvV62FjaJgzqORIhdFfq0nmj4%3D&reserved=0>.

*       Please upload any figures associated with your paper as individual TIF or EPS files with 300dpi resolution at resubmission; please read our figure guidelines for more information on our requirements: <https://gcc02.safelinks.protection.outlook.com/?url=http%3A%2F%2Fjournals.plos.org%2Fplosmedicine%2Fs%2Ffigures&data=05%7C02%7Cleila.abar%40nih.gov%7Cebb2ae5a8c8046e5f64008dd3ac4ac14%7C14b77578977342d58507251ca2dc2b06%7C0%7C0%7C638731338859719294%7CUnknown%7CTWFpbGZsb3d8eyJFbXB0eU1hcGkiOnRydWUsIlYiOiIwLjAuMDAwMCIsIlAiOiJXaW4zMiIsIkFOIjoiTWFpbCIsIldUIjoyfQ%3D%3D%7C0%7C%7C%7C&sdata=4u7kwtoa318viOc8LXKyvELKzBToq%2B7jSz05S4qiUZw%3D&reserved=0>. While revising your submission, please upload your figure files to the PACE digital diagnostic tool, <https://gcc02.safelinks.protection.outlook.com/?url=https%3A%2F%2Fpacev2.apexcovantage.com%2F&data=05%7C02%7Cleila.abar%40nih.gov%7Cebb2ae5a8c8046e5f64008dd3ac4ac14%7C14b77578977342d58507251ca2dc2b06%7C0%7C0%7C638731338859730784%7CUnknown%7CTWFpbGZsb3d8eyJFbXB0eU1hcGkiOnRydWUsIlYiOiIwLjAuMDAwMCIsIlAiOiJXaW4zMiIsIkFOIjoiTWFpbCIsIldUIjoyfQ%3D%3D%7C0%7C%7C%7C&sdata=LplOnNFWGdRa5uORg3w0EBnlzrF4X09fPHh0EzvUYHU%3D&reserved=0>. PACE helps ensure that figures meet PLOS requirements. To use PACE, you must first register as a user. Then, login and navigate to the UPLOAD tab, where you will find detailed instructions on how to use the tool. If you encounter any issues or have any questions when using PACE, please email us at [PLOSMedicine@plos.org](mailto:PLOSMedicine@plos.org).

*       Please ensure that the paper adheres to the PLOS Data Availability Policy (see <https://gcc02.safelinks.protection.outlook.com/?url=http%3A%2F%2Fjournals.plos.org%2Fplosmedicine%2Fs%2Fdata-availability&data=05%7C02%7Cleila.abar%40nih.gov%7Cebb2ae5a8c8046e5f64008dd3ac4ac14%7C14b77578977342d58507251ca2dc2b06%7C0%7C0%7C638731338859742449%7CUnknown%7CTWFpbGZsb3d8eyJFbXB0eU1hcGkiOnRydWUsIlYiOiIwLjAuMDAwMCIsIlAiOiJXaW4zMiIsIkFOIjoiTWFpbCIsIldUIjoyfQ%3D%3D%7C0%7C%7C%7C&sdata=J4LmZuzaarMT91M6D8M3VXzy%2BvZ9sJ6wGJ4c4vforJ8%3D&reserved=0)>, which requires that all data underlying the study's findings be provided in a repository or as Supporting Information. For data residing with a third party, authors are required to provide instructions with contact information (web or email address) for obtaining the data. Please note that a study author cannot be the contact person for the data. PLOS journals do not allow statements supported by "data not shown" or "unpublished results." For such statements, authors must provide supporting data or cite public sources that include it.

*       We expect all researchers with submissions to PLOS in which author-generated code underpins the findings in the manuscript to make all author-generated code available without restrictions upon publication of the work. In cases where code is central to the manuscript, we may require the code to be made available as a condition of publication. Authors are responsible for ensuring that the code is reusable and well documented. Please make any custom code available, either as part of your data deposition or as a supplementary file. Please add a sentence to your data availability statement regarding any code used in the study, e.g. "The code used in the analysis is available from Github [URL] and archived in Zenodo [DOI link]" Please review our guidelines at <https://gcc02.safelinks.protection.outlook.com/?url=https%3A%2F%2Fjournals.plos.org%2Fplosmedicine%2Fs%2Fmaterials-software-and-code-sharing&data=05%7C02%7Cleila.abar%40nih.gov%7Cebb2ae5a8c8046e5f64008dd3ac4ac14%7C14b77578977342d58507251ca2dc2b06%7C0%7C0%7C638731338859753356%7CUnknown%7CTWFpbGZsb3d8eyJFbXB0eU1hcGkiOnRydWUsIlYiOiIwLjAuMDAwMCIsIlAiOiJXaW4zMiIsIkFOIjoiTWFpbCIsIldUIjoyfQ%3D%3D%7C0%7C%7C%7C&sdata=wKyHn2zQu34O8VP6Uw%2Fjznn%2BW%2BrIS5ALti7WDoxLvtg%3D&reserved=0> and ensure that your code is shared in a way that follows best practice and facilitates reproducibility and reuse. Because Github depositions can
be readily changed or deleted, we encourage you to make a permanent DOI'd copy (e.g. in Zenodo) and provide the URL.

FORMATTING - GENERAL
*       Abstract: Please structure your abstract using the PLOS Medicine headings (Background, Methods and Findings, Conclusions). Please combine the Methods and Findings sections into one section.

*       At this stage, we ask that you include a short, non-technical Author Summary of your research to make findings accessible to a wide audience that includes both scientists and non-scientists. The Author Summary should immediately follow the Abstract in your revised manuscript. This text is subject to editorial change and should be distinct from the scientific abstract. Ideally each sub-heading should contain 2-3 single sentence, concise bullet points containing the most salient points from your study. In the final bullet point of 'What Do These Findings Mean?', please include the main limitations of the study in non-technical language. Please see our author guidelines for more information: <https://gcc02.safelinks.protection.outlook.com/?url=https%3A%2F%2Fjournals.plos.org%2Fplosmedicine%2Fs%2Frevising-your-manuscript%23loc-author-summary&data=05%7C02%7Cleila.abar%40nih.gov%7Cebb2ae5a8c8046e5f64008dd3ac4ac14%7C14b77578977342d58507251ca2dc2b06%7C0%7C0%7C638731338859765050%7CUnknown%7CTWFpbGZsb3d8eyJFbXB0eU1hcGkiOnRydWUsIlYiOiIwLjAuMDAwMCIsIlAiOiJXaW4zMiIsIkFOIjoiTWFpbCIsIldUIjoyfQ%3D%3D%7C0%7C%7C%7C&sdata=aKwbRiFkxOVW5GgWIegCPOjB0vmSuZFvz5U7w6X3Cbc%3D&reserved=0>.

*       Please express the main results with 95% CIs as well as p values. When reporting p values please report as p<0.001 and where higher as the exact p value p=0.002, for example. Throughout, suggest reporting statistical information as follows to improve clarity for the reader "22% (95% CI [13%,28%]; p</=)". Please be sure to define all numerical values at first use.

*       Please include page numbers and line numbers in the manuscript file. Use continuous line numbers (do not restart the numbering on each page).

*       Please cite the reference numbers in square brackets. Citations should precede punctuation.

*      PLOS Medicine requires that the de-identified data underlying the specific results in a published article be made available, without restrictions on access, in a public repository or as Supporting Information at the time of article publication, provided it is legal and ethical to do so. Please see the policy at

<https://gcc02.safelinks.protection.outlook.com/?url=http%3A%2F%2Fjournals.plos.org%2Fplosmedicine%2Fs%2Fdata-availability&data=05%7C02%7Cleila.abar%40nih.gov%7Cebb2ae5a8c8046e5f64008dd3ac4ac14%7C14b77578977342d58507251ca2dc2b06%7C0%7C0%7C638731338859777034%7CUnknown%7CTWFpbGZsb3d8eyJFbXB0eU1hcGkiOnRydWUsIlYiOiIwLjAuMDAwMCIsIlAiOiJXaW4zMiIsIkFOIjoiTWFpbCIsIldUIjoyfQ%3D%3D%7C0%7C%7C%7C&sdata=P4vvDqvZJXitOAWlla5Bks5ZIchm4igBibUeoC9otoc%3D&reserved=0>

and FAQs at

<https://gcc02.safelinks.protection.outlook.com/?url=http%3A%2F%2Fjournals.plos.org%2Fplosmedicine%2Fs%2Fdata-availability%23loc-faqs-for-data-policy&data=05%7C02%7Cleila.abar%40nih.gov%7Cebb2ae5a8c8046e5f64008dd3ac4ac14%7C14b77578977342d58507251ca2dc2b06%7C0%7C0%7C638731338859788008%7CUnknown%7CTWFpbGZsb3d8eyJFbXB0eU1hcGkiOnRydWUsIlYiOiIwLjAuMDAwMCIsIlAiOiJXaW4zMiIsIkFOIjoiTWFpbCIsIldUIjoyfQ%3D%3D%7C0%7C%7C%7C&sdata=YMEk4jzAyiYIPUYjr6wP0Zg3t8KgCpsaXarVo%2FfCu%2Bk%3D&reserved=0>

*      Please outline why your manuscript does not require ethics approval

FIGURES AND TABLES
*       Please provide titles and legends for all figures and tables (including those in Supporting Information files).

*       Please define all abbreviations used in each figure/table (including those in Supporting Information files).

*       Please consider avoiding the use of red and green in order to make your figure more accessible to those with color blindness.

SUPPLEMENTARY MATERIAL
*       Please note that supplementary material will be posted as supplied by the authors. Therefore, please amend it according to the relevant comments outlined here.

*       Please cite your Supporting Information as outlined here: <https://gcc02.safelinks.protection.outlook.com/?url=https%3A%2F%2Fjournals.plos.org%2Fplosmedicine%2Fs%2Fsupporting-information&data=05%7C02%7Cleila.abar%40nih.gov%7Cebb2ae5a8c8046e5f64008dd3ac4ac14%7C14b77578977342d58507251ca2dc2b06%7C0%7C0%7C638731338859800065%7CUnknown%7CTWFpbGZsb3d8eyJFbXB0eU1hcGkiOnRydWUsIlYiOiIwLjAuMDAwMCIsIlAiOiJXaW4zMiIsIkFOIjoiTWFpbCIsIldUIjoyfQ%3D%3D%7C0%7C%7C%7C&sdata=L5CViv8GEld3vJWgwdud1fuUpWt01q1Q38xMgbj%2BXn4%3D&reserved=0>

REFERENCES
*       PLOS uses the numbered citation (citation-sequence) method and first six authors, et al.

*       Please ensure that journal name abbreviations match those found in the National Center for Biotechnology Information (NCBI) databases (<http://www.ncbi.nlm.nih.gov/nlmcatalog/journals>), and are appropriately formatted and capitalised.

*       Where website addresses are cited, please include the complete URL and specify the date of access (e.g. [accessed: 12/06/2023]).

*       Please also see <https://gcc02.safelinks.protection.outlook.com/?url=https%3A%2F%2Fjournals.plos.org%2Fplosmedicine%2Fs%2Fsubmission-guidelines%23loc-references&data=05%7C02%7Cleila.abar%40nih.gov%7Cebb2ae5a8c8046e5f64008dd3ac4ac14%7C14b77578977342d58507251ca2dc2b06%7C0%7C0%7C638731338859812522%7CUnknown%7CTWFpbGZsb3d8eyJFbXB0eU1hcGkiOnRydWUsIlYiOiIwLjAuMDAwMCIsIlAiOiJXaW4zMiIsIkFOIjoiTWFpbCIsIldUIjoyfQ%3D%3D%7C0%7C%7C%7C&sdata=Qqxck2ODihWJxlqSMqNSNBLyTpSm04bSc%2F%2BbPo5ohe0%3D&reserved=0> for further details on reference formatting.

RCTs

*       PLOS Medicine requires that all trials be prospectively registered in one of registries recognized by WHO. Please ensure that study registration details are included in the Methods section.

*       Please structure the Methods section using the following sub-headings: Study design and participants, Randomization and masking, Procedures, Outcomes, Statistical analysis.

*       Please ensure that all prespecified outcomes (primary, secondary, and exploratory) are listed in the Methods/Outcomes section and indicate whether there are outcomes that are not presented in the current report.

*       Please specify the dates (Month Day, Year) during which study enrollment and follow up occurred.

*       Please include absolute numbers wherever you report percentages; eg, n/N (%)

*       Please present the safety data for the study including numbers of specific events and whether or not adverse events are thought to be related to treatment. AEs should be reported in the abstract, per CONSORT and CONSORT-Harms.

*       Please complete the CONSORT checklist (<https://gcc02.safelinks.protection.outlook.com/?url=https%3A%2F%2Fwww.equator-network.org%2Freporting-guidelines%2Fconsort%2F&data=05%7C02%7Cleila.abar%40nih.gov%7Cebb2ae5a8c8046e5f64008dd3ac4ac14%7C14b77578977342d58507251ca2dc2b06%7C0%7C0%7C638731338859824032%7CUnknown%7CTWFpbGZsb3d8eyJFbXB0eU1hcGkiOnRydWUsIlYiOiIwLjAuMDAwMCIsIlAiOiJXaW4zMiIsIkFOIjoiTWFpbCIsIldUIjoyfQ%3D%3D%7C0%7C%7C%7C&sdata=FVgcY2%2BjmzdbViFfpIwVNosITxYZm2oB1QSEDdpIH7Q%3D&reserved=0>) and ensure that all components of CONSORT are present in the manuscript, including how randomization was performed, allocation concealment, blinding of intervention, definition of lost to follow-up, power statement. When completing the checklist, please use section and paragraph numbers, rather than page numbers.

*       Please report your abstract according to CONSORT for abstracts, following the PLOS Medicine abstract structure (Background, Methods and Findings, Conclusions) <https://gcc02.safelinks.protection.outlook.com/?url=https%3A%2F%2Fwww.equator-network.org%2Freporting-guidelines%2Fconsort-abstracts%2F&data=05%7C02%7Cleila.abar%40nih.gov%7Cebb2ae5a8c8046e5f64008dd3ac4ac14%7C14b77578977342d58507251ca2dc2b06%7C0%7C0%7C638731338859836023%7CUnknown%7CTWFpbGZsb3d8eyJFbXB0eU1hcGkiOnRydWUsIlYiOiIwLjAuMDAwMCIsIlAiOiJXaW4zMiIsIkFOIjoiTWFpbCIsIldUIjoyfQ%3D%3D%7C0%7C%7C%7C&sdata=CyjgVdtvTWz1yHWEN1NPzkjwC0d71aBwXTXhdkLtGmg%3D&reserved=0>

*       If your trial had to undergo important modifications in response to extenuating circumstances, please complete the CONSERVE-CONSORT checklist and provide in your Supporting Information; (<https://gcc02.safelinks.protection.outlook.com/?url=https%3A%2F%2Fwww.equator-network.org%2Freporting-guidelines%2Fguidelines-for-reporting-trial-protocols-and-completed-trials-modified-due-to-the-covid-19-pandemic-and-other-extenuating-circumstances-the-conserve-2021-statement%2F&data=05%7C02%7Cleila.abar%40nih.gov%7Cebb2ae5a8c8046e5f64008dd3ac4ac14%7C14b77578977342d58507251ca2dc2b06%7C0%7C0%7C638731338859846883%7CUnknown%7CTWFpbGZsb3d8eyJFbXB0eU1hcGkiOnRydWUsIlYiOiIwLjAuMDAwMCIsIlAiOiJXaW4zMiIsIkFOIjoiTWFpbCIsIldUIjoyfQ%3D%3D%7C0%7C%7C%7C&sdata=jKsBptwKq9CwCgGm4RArKdVE2QZwtRj7Jd5b9Qoohok%3D&reserved=0>). When completing the checklist, please use section and paragraph numbers, rather than page numbers.

*       In keeping with our commitment to Open Science, please include the study protocol document and analysis plan (including any amendments) as Supporting Information to be published with the manuscript if accepted.

*       Please note that PLOS Medicine requires prospective, public registration of a data sharing plan (as part of mandatory clinical trials registration) for all clinical trials that began enrollment on or after January 1, 2019, in accordance with ICMJE requirements.

In compliance with data protection regulations, you may request that we remove your personal registration details at any time.  (Use the following URL: <https://gcc02.safelinks.protection.outlook.com/?url=https%3A%2F%2Fwww.editorialmanager.com%2Fpmedicine%2Flogin.asp%3Fa%3Dr&data=05%7C02%7Cleila.abar%40nih.gov%7Cebb2ae5a8c8046e5f64008dd3ac4ac14%7C14b77578977342d58507251ca2dc2b06%7C0%7C0%7C638731338859860564%7CUnknown%7CTWFpbGZsb3d8eyJFbXB0eU1hcGkiOnRydWUsIlYiOiIwLjAuMDAwMCIsIlAiOiJXaW4zMiIsIkFOIjoiTWFpbCIsIldUIjoyfQ%3D%3D%7C0%7C%7C%7C&sdata=u12Dlj2mUdoHU4GuqSV2ILp9fqoUpxaCx1sr8Yabi2o%3D&reserved=0)>. Please contact the publication office if you have any questions.
CAUTION: This email originated from outside of the organization. Do not click links or open attachments unless you recognize the sender and are confident the content is safe.
